# Exercise-Induced Bronchoconstriction in Children: A Comparison between Athletes and Non-Athletes

**DOI:** 10.3390/healthcare11091349

**Published:** 2023-05-08

**Authors:** Kamila Malewska-Kaczmarek, Daniela Podlecka, Tymoteusz Mańkowski, Joanna Jerzyńska, Iwona Stelmach

**Affiliations:** 1 Korczak Pediatric Center, Department of Pediatrics and Allergology, Medical University of Lodz, al. Pilsudskiego 71, 92-328 Lodz, Poland; 2 Department of Radiology, Nicolaus Copernicus Regional Multi-Specialty, Oncology and Trauma Centre in Lodz, 93-513 Lodz, Poland; 3 Poddębice Health Care Centre, 99-200 Poddębice, Poland

**Keywords:** EIB, adolescent athletes, schoolchildren

## Abstract

Exercise-induced bronchoconstriction (EIB) is a dysfunction of the respiratory tract consisting of transient airflow obstruction. This study is a retrospective analysis of two prospective studies concerning EIB symptoms in two adolescent populations. Our study group included 400 non-athletes and 101 athletes. Due to the similarity of indoor exercise conditions, an analysis was performed on the basis of where training took place. The study aims to assess the EIB prevalence in the following groups of adolescent children: non-athletes and athletes. In “indoor” athletes, the EIB prevalence was 22.4%. Among non-athletes, EIB was diagnosed in 10.2% (*p* = 0.007). A history of asthma was found in 6.5% of non-athletes and 29.3% of indoor athletes (*p* < 0.001). The incidence of EIB without asthma was higher in indoor athletes (14.6%) than in non-athletes (9.9%). Athletes achieved higher mean values in forced expiratory volume in one second (FEV_1_), forced vital capacity (FVC), peak expiratory flow (PEF), and maximum expiratory flow rate at 25% (MEF_25_) parameters. In the group of non-athletes, higher results were observed in forced expiratory volume in one second % of vital capacity (FEV_1_%VC), MEF_50_, and MEF_75_. The findings of the study present the complexity of the EIB diagnosis among children training in an indoor environment.

## 1. Introduction

Maintaining good health means maintaining a healthy lifestyle. One way to manage good health is regular physical activity, especially at a young age. Evidence-based guidelines and healthcare systems recommend routine physical activity as the best way to prevent diseases [1]. However, exercise may not be accessible to everyone. One of the factors that may particularly limit physical fitness may be respiratory symptoms during exercise. Exercise-induced bronchoconstriction (EIB) is defined as the coexistence of at least two of the following symptoms: dyspnea, cough, wheezing, shortness of breath and chest pain, combined with the decrease in forced expiratory volume in one second (FEV_1_). Perception of these symptoms may restrain children’s ability and willingness to exercise [2,3].

EIB is a dysfunction of the respiratory tract consisting of transient airflow obstruction. It can be observed both in healthy and asthmatic patients. Typically, symptoms occur 5–15 min during or after physical activity. Acute airway narrowing due to exercise may influence the quality and length of training. Particularly, it is observed in athletes in whom EIB prevalence varies between 30% and 70% [4]. The results of our previous study revealed a 28% prevalence of EIB in adolescent athletes from the Lodz Metropolitan Area, Poland [5]. Decreased athletic performance may be a result of frequent exposure to EIB triggers. It can be observed especially in those exposed to chlorine compounds (e.g., swimmers) and cold air (e.g., cross-country skiers, ice hockey players and skaters). Intense exercise leads to a higher degree of ventilation and increased breathing capacity. This may influence increased water loss from the airways and a decrease in mucosal temperature. A large volume of inspired cold air leads to dehydration of respiratory mucous membranes, vasoconstriction and subsequent hyperemia. All these factors, including osmotic and thermal changes of the bronchial mucosa, combined with irritation by airborne pollutants and excessive mucus production, may trigger bronchoconstriction [4,6].

EIB also occurs in a general pediatric population in which physical activity is not as expressed as in the athlete population. The prevalence of EIB in this group ranges from 3% to 35%, depending on a study [6,7]. Furthermore, particular attention should be paid to children with asthma diagnosis. In the recent review, authors state that EIB prevalence in asthmatic children varies between 40 and 90% [3]. In the past, bronchoconstriction during exercise in asthmatic patients was called exercise-induced asthma (EIA). The latest research distinguishes between EIB with asthma (EIB_A_) and EIB without asthma (EIB_WA_) [4,6,8]. However, some research revealed that asthma risk in children is influenced by the level of physical activity. The authors demonstrated that increased moderate-to-vigorous physical activity (MVPA) is associated with lower asthma risk. This was especially evident in children with low activity levels. Moreover, the high activity level in children is associated with greater asthma risk [9]. For this reason, there should be greater awareness of EIB and asthma in young athletes. 

The aim of the study was to compare the general population of adolescent non-athletes adolescent athletes in terms of EIB and asthma prevalence.

## 2. Materials and Methods

The study is a retrospective analysis of two prospective studies involving EIB symptoms in two adolescent groups.

The first prospective study included 400 non-athletes aged 13–18. The research took place between February 2013 and December 2014 and was conducted in seven randomly selected public schools in Lodz. A total of 400 patients were randomly selected from the dataset of 1002 clinical trial participants who took part in one physical education (PE) session. The study group was labeled as “non-athletes”, with an average amount of physical exercise, including PE lessons at school (as it is required for healthy children a minimum of three times per week for approximately 45 min). Both before and after the PE lesson, each of the students was examined by a doctor, including measurements of vital signs. Medical personnel took a detailed history of each patient, including the diagnosis of atopy. Exclusion criteria from the study included respiratory diseases (other than asthma), including recent infection (four weeks before the study), orthopedic or neurological disorders and cardiac diseases. All detailed data analyzed during this study are included in the previously published article (the study was approved by the Medical Ethical Committee of the Medical University of Lodz No. RNN/160/12/KE). [10].

The second prospective study included 101 athletes aged 12–18. A prospective study was conducted at the Department of Pediatrics and Allergy, Korczak Pediatric Center in Lodz, between March 2019 and January 2022. Children, who trained a minimum of four times per week for approximately 90 min, were considered “athletes” and invited to the study. The athletic group practiced the following sports: football, swimming, horse riding, cycling, athletics, tennis, dance, martial arts, floorball, basketball, volleyball, and handball. After obtaining a written consent form from parents/legal guardians and patients, the athletic group underwent a standardized exercise challenge on an electronically controlled treadmill. Beforehand, the medical personnel collected a detailed history of each athlete. Before and after the tests, each patient underwent a physical examination. Exclusion criteria were as follows: lack of cooperation with the patient, chronic respiratory system disease (other than asthma) and the presence of contraindications for the tests (as determined by the Polish Society of Allergology). All the analyzed data concerning this study is included in the previously published article (the study was approved by the Medical Ethical Committee of the Medical University of Lodz No. RNN/303/17/KE) [5].

Both studies analyzed EIB occurrence during physical activity. A group of children from public schools participated in a PE lesson, and those from sports schools were compared. Due to the similarity of indoor exercise conditions, an analysis was made on the basis of training location. Both non-athletes and athletes participating in the study trained in similar conditions (sports hall). Among the athletes, a group practicing indoors was selected and called “indoor athletes”.

### 2.1. Spirometry

In the group of non-athletes, spirometry was performed using a portable spirometer (Spirolab III; MIR SRL, via del Maggiolino 125, 00155 Roma, Italy). If three technically acceptable FEV_1_ measurements were performed, the highest FEV_1_ was selected. FEV_1_ measurements were taken before and immediately after the 45 min PE lesson. All the details in a previously published article [10].

Among the group of athletes, spirometry was performed using a Master Screen unit (Erich Jaeger Gmbh-Hochberg, Strassheimer Str. 10, 61169 Friedberg, Germany). If three forced expirations preceded by maximal inspirations were technically acceptable, the results were utilized for analysis. The details of the research are written in a previously published article [5].

The test results were described as absolute values and related to the normal range. A normal value was described as if it was greater than or equal to the lower limit of normal in the reference population. Then the result was presented as a percent predicted value.

All measurements were made in accordance with the American Thoracic Society/European Respiratory Society (ATS/ERS) guidelines [11,12].

### 2.2. Exercise Challenge

The exercise challenge in the group of non-athletes consisted of two FEV_1_ measurements performed using a portable spirometer (non-standardized exercise challenge). Measurements were obtained before and after a 45 min indoor PE lesson. Vital signs were also measured. Each physical exercise was preceded by a 10 min warm-up. The research was conducted in the sports hall, with similar exercise intensity for each participant. If the difference between FEV_1_% after and FEV_1_% before the exercise was equal to or more than 10%, the physician made the diagnosis of EIB. Patients with symptoms of EIB during or after PE lessons (dyspnoea, wheezing on auscultation, cough) were also recorded [10].

The exercise challenge among adolescent athletes was a standardized test, according to the recommendations for provocative tests in allergy by the ATS/ERS [11]. It was conducted on an electronically controlled treadmill (Kettler, Trisport AG, Boesch 67 CH-6331 Huenenberg, Switzerland). The following variables were monitored during the challenge: the test room temperature (should be less than 25 °C) and the humidity (should be less than 50%). Furthermore, the patient’s heart rate was monitored by a pulse meter, an integral component of the electronic treadmill. Twenty and five minutes before the exercise challenge, each patient had a spirometry test. Each challenge was preceded by a two-minute burn-up exercise consisting of a gradual increase in treadmill speed. The incline of the treadmill was set at 3 degrees. If the heart rate level reached 95% of the maximum calculated result for a particular person, a six-minute exercise challenge was performed. Spirometry was performed again at 1, 3, 6, 10, 15, and 20 min after the exercise test. The diagnosis of EIB was made if the decline in FEV_1_% was greater or equal to 10% above baseline. Patients with symptoms of EIB (wheezes, dyspnoea) during or directly after the exercise challenge were also recorded [5].

### 2.3. Statistical Analysis

Categorical variables were depicted using integer numbers and percentages. Numerical traits were described using mean, median, standard deviation and minimum-maximum values. The normality of distribution was assessed using the Anderson–Darling test. The homogeneity of variances was appraised by using Levene’s test. Statistical significance in the numerical variables was tested by using a multifactor ANOVA without replication for normally distributed variables. Otherwise, generalized linear models with robust standard errors were fitted for non-normally distributed traits. For categorical variables, the Person Chi-squared test of independence was performed for descriptive between-group purposes. When assessing between-group differences in the spirometry results, multifactor stepwise regression models were fitted, controlling for the occurrence of selected disorders (pre-existing cough, asthma, EIB symptoms during the test). The clinical accuracy of diagnostic procedures applied in both study groups was assessed using the Mantel–Haenszel Chi-square test. A level of *p* < 0,05 was deemed statistically significant. All the statistical procedures were performed by using Minitab^®^, release 21.1 (Minitab, LLC, State College, PA, USA).

The sampling frame of the comparison group was a dataset of 1002 non-athletes. A Microsoft Excel spreadsheet was used to randomize these subjects using the RANDOM() function. Following the recommendations of other researchers, a decision was made to increase the target group size by a factor of four. The column with the generated random numbers was sorted in ascending order, and the first 400 records were selected. These records became part of the comparison (reference) group [13].

## 3. Results

**Figure 1:**
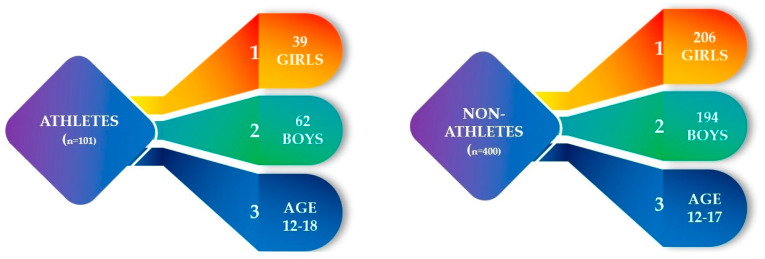
Baseline characteristics of the study cohort (n = 501).

The study group included 400 non-athletes and 101 athletes from Lodz. All details concerning the patients are shown in Figure 1.

Indoor athletes (n = 58) were selected from the group of athletes. In this group, the EIB prevalence was 22.4% (n = 13). Among non-athletes, an EIB diagnosis was made in 10.2% (n = 41) (*p* = 0.007). A history of asthma was found in 6.5% of non-athletes and 29.3% of indoor athletes (*p* < 0.001). Among non-athletes, 17.5% reported coughing during exercise, compared to 24.13% of athletes, but these results were not statistically significant. Among non-athletes, only 5.7% had symptoms during the exercise challenge. In the indoor athletic group, 17.2% of patients reported symptoms during the exercise test (*p* = 0.011). All details concerning indoor athletes and non-athletes are shown in Table 1.

### 3.1. Spirometry Characteristics

The differences in spirometry results between indoor athletic and non-athletic groups were observed in the study. As was expected, athletes achieved higher mean values in FEV_1_ (*p* < 0.001), forced vital capacity (FVC) (*p* < 0.001), peak expiratory flow (PEF) (*p* < 0.001), and maximum expiratory flow rate at 25% (MEF_25_) (*p* < 0.001) parameters. In the group of non-athletes, higher results were observed in FEV_1_%VC (*p* = 0.004), MEF_50_ (*p* = 0.348), and MEF_75_ (*p* = 0.011). All the details concerning spirometry results are shown in Table 2.

### 3.2. Indoor Athletes and Non-Athletes

We analyzed athletes performing indoor activities considering the different environmental factors influencing EIB and spirometry. Details concerning indoor athletes are shown in Table 3.

**Figure 2:**
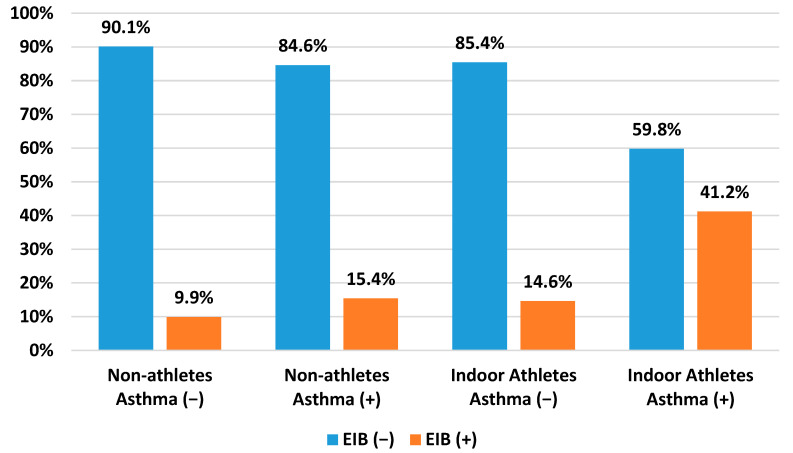
Comparison between EIB and asthma among non-athletes and indoor athletes.

The incidence of EIB_WA_ was higher in indoor athletes (14.6%) (*p* = 0.027) compared to non-athletes (9.9%) (*p* = 0.372). Furthermore, more children had asthma without EIB in the group of non-athletes (84.6%) compared to the athletic group (59.8%). EIB_A_ was more frequent in indoor athletes (41.2%) than in non-athletes (15.4%). All the details concerning EIB and asthma are shown in Figure 2. The comparison between both groups was statistically significant (*p* < 0.001).

**Figure 3:**
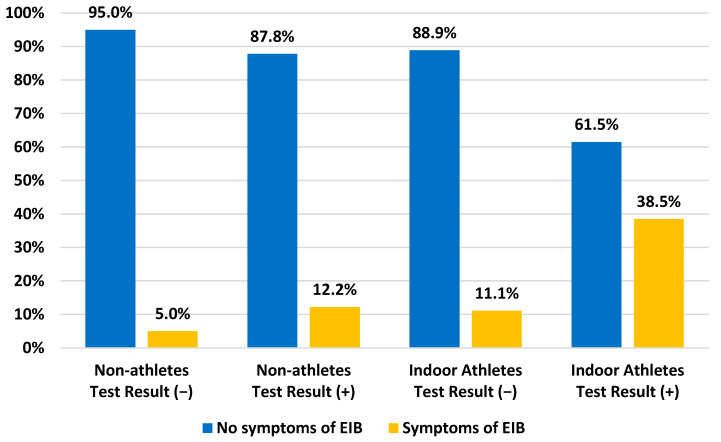
Comparison between symptoms of EIB and the result of exercise challenge among non-athletes and indoor athletes.

Patients with no EIB symptoms during the tests but with positive exercise challenges were found in 61.5% and 87.8% of cases in indoor athletes (*p* = 0.03) and non-athletes (*p* = 0.061), respectively. In contrast, 11.1% of indoor athletes and 5% of non-athletes had EIB symptoms during the exercise challenge, but the test result was negative. All the details concerning symptoms of EIB and the results of the exercise challenge are shown in Figure 3. The differences between both groups were statistically significant (*p* = 0.011).

Pre-existing cough without EIB diagnosis was observed in 57.1% of indoor athletes (n = 8) (*p* = 0.035) and 78.6% of non-athletes (n = 55) (*p* < 0.001). Comparison between both groups was statistically not significant (*p* = 0.485). 

## 4. Discussion

To our knowledge, this is the first research evaluating EIB in a sample of adolescent non-athletes and athletes training indoors.

In the previous research, we observed that the group of adolescents training indoors (sports hall) differed from other study participants. Firstly, we demonstrated that compared to outdoor athletes, indoor athletes achieved lower spirometry parameters. We then argued that more indoor than outdoor athletes were diagnosed with EIB [5]. This refers to our previous research, and this is a short explanation of why we decided to address indoor athletes. This topic remained our subject of interest and aimed to evaluate the differences between indoor athletes and non-athletes.

### 4.1. Comparison between Indoor Athletes and Non-Athletes

Our study revealed an EIB prevalence in 22.4% of indoor athletes. This is a significant result, especially since the prevalence of less athletic children training under the same conditions is 10.2%. Research by other scientists also indicates the significant problem of EIB in young athletes. According to meta-analysis by de Aguiar et al., the EIB prevalence in young athletes was 15% [14]. In the study by Jonckheere et al., 24% of early-career athletes had EIB. In the same study, EIB prevalence in young basketball players was 27.3% and in swimmers, 33.3% [15]. This raises the question of why EIB is more common in adolescent athletes than non-athletes. One of the possible reasons may be due to the pathophysiology of the exercise itself. Increased ventilation during the exercise promotes dehydration of the airways and loss of temperature, which may lead to bronchial muscle contraction. Moreover, these changes result in osmotic change and mast cell degranulation, which further exacerbates bronchospasms [7,16]. Recent literature outlines that EIB’s underlying cause in athletes is the result of a combination of factors. Intense, prolonged exercise leads to chronic exposure to air pollutants, airborne allergens, and dry air stressors. Additionally, sensory nerve activation with mast cell mediator release may influence proinflammatory response and bronchoconstriction. Atchley et al. suggested that compared to non-athletes, athletes have increased parasympathetic and reduced sympathetic neuronal activity. Due to intense exercise, athletes may have chronic increased vagus nerve stimulation. Vagal activation promotes the stimulation of parasympathetic nerves in the bronchi, which in turn lowers the threshold for bronchoconstriction [6]. Other researchers also indicated that epithelial damage may affect the development of EIB in young athletes. Jonckheere et al. found increased levels of serum club cell protein 16 (CC16) and sputum uric acid in early-career athletes. In addition, researchers found a correlation between higher CC16 concentrations and greater FEV_1_ decline. These findings may indicate the important role of epithelial damage in EIB development [15].

Concomitantly, Stelmach et al. provided significant evidence of environmental factors (e.g., humidity and barometric pressure) and cat dander allergens sampled at the sports hall that may influence bronchoconstriction in children. Our study did not include an analysis of these factors, but we relied on evidence-based data. The authors of the research also indicate that exposure to mold in school indoor environment may play a significant role in asthma-related symptoms [17]. These results were consistent with our previous findings. We argued that atopy to grass, trees, and mold in adolescent athletes might influence FEV_1_ decline in exercise challenges [5].

EIB, in general, usually appears 5–15 min during or after exercise, with a peak after 10 min. Symptoms usually resolve after 60 min [4,7]. Until now, it was thought that EIB might be connected to years devoted to training athletes. On the other hand, some research indicates that EIB in athletes may be present already at the beginning of their careers. This particularly affects early-career athletes, who may either deny EIB symptoms or give up training. In the research by Jonckheere et al., EIB prevalence in athletes aged 12–13 years old was 24.5% [15]. For this reason, attention should be drawn to the special role of screening diagnosis to identify those who may have EIB [4].

In summary, all these variables may affect EIB occurrence in adolescent athletes. High-intensity exercise with high minute ventilation of the lungs leads to epithelial injury. Simultaneously, environmental factors (e.g., indoor environment) play a significant role in bronchoconstriction. This may explain why indoor athletes are more likely to develop EIB.

### 4.2. Asthma

In the pathogenesis of EIB, experts underline the differences between EIB_WA_ and EIB_A_. In the case of EIB_WA_, airway epithelial injury plays a major role in disease development [6]. According to this EIB classification, we discovered a prevalence of EIB_WA_ in 14.6% of our athletic and 9.9% of our non-athletic groups.

Zeiger et al. described that EIB_WA_ prevalence in athletes depends on the duration and intensity of physical training. According to their research, EIB_WA_ is associated with more strenuous endurance sports. Swimming and winter sports are especially fraught with a high risk of EIB [18,19], as observed in our research. This also explains the higher incidence of adolescent EIB_WA_ in athletes than in non-athletes.

EIB in asthmatic patients results from classic eosinophilic and mast-cell-dependent inflammation [6]. According to Bonini et al., in a general population, EIB ranges from 3.7% to 54.8% of patients with asthma [1]. Our results are in accordance with this study, pointing out 15.4% EIB_A_ occurrence in non-athletes from the Lodz Metropolitan Area. Furthermore, we observed that 41.2% of indoor adolescent athletes are within this range. The following question arises: why is the prevalence of EIB_A_ in adolescent athletes higher than in the general population? Asthma in adolescent athletes poses a significant problem. Del Giacco states that asthma prevalence in adolescent athletes varies between 12 and 38%. In addition, the authors suggest that a diagnosis of EIB/EIB_A_ may pose certain difficulties, particularly in adolescent athletes, due to the possible absence of symptoms or total disregard of symptoms by such patients. 

First of all, adolescent asthmatic patients perceive themselves as completely healthy, leading them to deny the symptoms of the disease. The adolescent period is a difficult time for a young person, associated with peer and family pressure and underestimation of the disease. Furthermore, adolescents make the same attempts as their peers—smoking cigarettes and risky behavior, which can contribute to a lack of treatment, exacerbations and worsening of asthma control. Moreover, studies show that the most common treatment in this group is bronchodilators (β_2_-agonists). This raises several problems due to the anti-doping regulations by which athletes are restricted. For this reason, some asthmatic patients may not want to use such treatment. On the other hand, there is a group of athletes who may abuse this medication. Therefore, both coaches and athletes should be trained in proper asthma management [19]. 

Studies investigating EIB_A_ in competitive athletes suggest that athletes with asthma are at a higher risk of ventilatory-related injuries caused by exercise. Additionally, they emphasize that increased ventilation combined with noxious environmental factors leads to increased airway inflammation and asthma risk. Nonetheless, it is believed that physical activity and appropriate treatment benefit asthma patients. This has allowed asthmatic athletes to perform sports even at the elite level [9,20].

Atchley et al. note that it is essential to carefully distinguish between EIB_WA_ and EIB_A_. This especially applies to EIB_A_ because there are several indications in favor of this diagnosis. If a patient with EIB (1) has symptoms in the night or early morning, (2) has symptom exacerbations during an infection, after inhalation of irritants or allergens, or (3) has a certain subset of symptoms, they should be evaluated for asthma [6].

### 4.3. Pre-Existing Chronic Cough

There are two phenotypes of asthma among professional athletes. The first is associated with a history of early childhood asthma. This type is connected with atopy, which occurs in early childhood. The second phenotype is related to athletes who train regularly. In this category, due to repeated, heavy exercise in preparation for competitions, athletes who experience epithelial injury may develop asthma. These professionals do not necessarily have typical asthma symptoms but may experience bronchial hyperresponsiveness. This results in chronic cough and excess mucous production.

In addition, asthma may be the result of repeated excessive exercise or viral infections [21]. Our current findings are in accordance with this statement, as 42.9% of adolescent indoor athletes had experienced pre-existing cough and EIB, compared to 21.4% of non-athletes. Furthermore, cough is one of the typical symptoms of EIB [7]. In our previous study, we indicated that cough may be the strongest predictor of EIB [10]. Our findings provide significant evidence of a correlation between EIB and cough. However, a large number of children without EIB diagnosis, both non-athletes (78.6%) and athletes (57.1%) report pre-existing cough. It is, therefore, important to rule out post-infection hyperreactivity. Occasionally a cough may persist up to three months after the infection [22].

Are there any other diseases underlying cough in children? In the differential diagnosis of EIB and asthma, there are other conditions to consider. Primarily, the aforementioned bronchial hyperresponsiveness can cause cough symptoms after intensive exercise. Moreover, exercise-induced laryngeal dysfunction should be considered in the differential diagnosis. Symptoms of this disorder are exclusive to exercise. Likewise, swimming-induced pulmonary edema should be considered for swimmers [7]. It is worth noting that gastroesophageal disease (GERD) may also manifest in chronic cough [22]. All of these disorders were taken into consideration in the analysis and differential diagnosis of our study group, but further research is required to assess the causes of chronic cough in the Lodz metropolitan area population.

### 4.4. Spirometry in Indoor Athletes and Non-Athletes

Spirometry is known as a gold standard among pulmonary function tests. Durmic et al. evaluated spirometry results among athletes and indicated that athletes achieved higher values than predicted [23]. We were able to reproduce similar results in our study. Our data provide significant evidence that competitive adolescent athletes achieve higher spirometry results in FEV_1_, FVC, MEF_25_ and PEF than non-athletes.

Conversely, the results of MEF_50_ and MEF_75_ were higher in non-athletes, while in athletes, MEF_50_ and MEF_75_ were still within normal range. These values are nicknamed “the quiet zone”, which represents small airway function and dysfunction. In the study by Rong et al., parameters responsible for small airway dysfunction in adult endurance athletes and swimmers were lower than 80% of the predicted values [24]. On the other hand, a study by Lipworth et al. suggests that in the interpretation of spirometry, the main difficulty with parameters MEF_50_ and MEF_75_ is that they are less easily reproducible than FEV_1_. The authors further suggest that this result is volume-dependent, which may confound the comparison between athletes and non-athletes [25].

It is important to remember that normal spirometry results do not exclude asthma diagnoses. If a patient is suspected of having airflow obstruction, a bronchodilator reversibility test should be considered [26].

### 4.5. Limitations and Study Strengths

The limitations of our study should be analyzed in the context of restrictions imposed during the SARS-CoV-2 pandemic. First and foremost, we examined a relatively small sample of adolescent athletes. Consequently, such data may drastically differ from that of a larger group of non-athletes. However, one should consider that far fewer children train on an athletic level than the vast majority that exercise during compulsory PE lessons at school. Additionally, we did not analyze the quality of life and demographics in both groups, which itself could be a valuable source of data for analysis; this will be the subject of a future study. In comparison between both study groups, it should be noted that FEV_1_ measurements taken during PE lessons are non-standardized exercise challenges (compared to standardized exercise challenges on a treadmill). Furthermore, we did not assess factors such as barometric pressure, humidity or allergens in the sports hall. Taking into account these values, this will be the subject of our future research.

Among the adolescent athletes, there was a broad involvement in a range of training sports, which may be an advantage or disadvantage of the research model. Each sport introduces varying degrees of exertion in its own distinct patterns (e.g., differing duration of matches, the proportion of active involvement by each team player, primary muscles involved, breathing technique, body temperature, and rapidity of movements), all of which may introduce a new set of confounding factors between types of sports. On the other hand, a model that takes into account detailed differences between each sport may artificially exacerbate results between the athletic and non-athletic groups. For example, novice swimmers need to carefully develop proper breathing techniques before intense swimming for longer time periods. Such a study model may have also made it more difficult to compare data between indoor and outdoor groups. Therefore, by choosing to study several sports simultaneously, we have attempted to “randomize” the confounding factors of each sport.

One of the major strengths of our research is the analysis of non-athletes. These findings may better reflect the values of the general population of school children. Efforts in this research to lower the occurrence of EIB among young professional athletes should improve their quality of life in the long term.

## 5. Conclusions

In conclusion, the findings of the study portray the complexity of a proper EIB diagnosis among children training in an indoor environment. Many variables, such as environment and asthma comorbidity, should be taken into account during EIB diagnosis and differentiation. EIB and asthma pose several challenges, especially among adolescents. Chronic absence during a PE lesson or during athletic training may represent a common consequence of EIB symptoms. For these reasons, a stronger collaboration between healthcare professionals and young athletes should improve both emotional and physical well-being for a lifetime of sports enjoyment.

## Figures and Tables

**Table 1:** Details concerning non-athletes and indoor athletes.

	Non-Athletes (n = 400)	Indoor Athletes (n = 58)	*p*
History of asthma (n = 43)	26 (6.5%)	17 (29.3%)	<0.001
Nonasthma (n = 415)	374 (93.5%)	41 (70.7%)	
EIB prevalence (n = 54)	41 (10.2%)	13 (22.4%)	=0.007
EIB symptoms during exercise challenge (n = 33)	23 (5.7%)	10 (17.2%)	=0.011
Children reporting pre-existing cough (n = 84)	70 (17.5%)	14 (24.13%)	=0.485

**Table 2:** Spirometry results divided by the study group (n = 458).

Variables	Statistical Parameter				*p*
	Non-Athletes (n = 400)		Indoor Athletes (n = 58)		
	M	SD	M	SD	
FEV_1_ * (%)	99.03	13.23	106.05	15.91	<0.001
FVC ** (%)	93.74	13.72	105.37	13.82	<0.001
FEV_1_%VC *** (%)	106.31	10.06	100.91	7.52	=0.004
PEF **** (%)	85.65	17.78	94.71	17.45	<0.001
MEF_25_ ***** (%)	84.73	17.81	90.40	29.29	<0.001
MEF_50_ (%)	100.82	23.32	93.16	23.40	=0.348
MEF_75_ (%)	113.69	34.32	97.14	20.42	=0.011

(Multivariate stepwise models were fitted by study group. M—mean, SD—standard deviation). * Forced expiratory volume in one second. ** Forced vital capacity. *** Forced expiratory volume in one second % of vital capacity. **** Peak expiratory flow. ***** Maximal expiratory flow at 25%, 50% and 75% of vital capacity.

**Table 3:** Characteristics of indoor athletes (n = 58).

Variables		
	Tennis, n (%)	2 (3.4)
	Dance, n (%)	4 (6.9)
	Athletics, n (%)	4 (6.9)
	Cycling, n (%)	4 (6.9)
Indoor athletes	Martial arts, n (%)	8 (13.8)
	Floorball, n (%)	1 (1.7)
	Basketball, n (%)	10 (17.2)
	Volleyball, n (%)	2 (3.4)
	Handball, n (%)	2 (3.4)
	Swimming, n (%)	21 (36.2)

## Data Availability

Not applicable.
